# Treatment of depression in cancer patients

**DOI:** 10.3747/co.2007.146

**Published:** 2007-10

**Authors:** G. Rodin, M. Katz, N. Lloyd, E. Green, J.A. Mackay, R.K.S. Wong

**Affiliations:** * Department of Psychosocial Oncology and Palliative Care, Princess Margaret Hospital, Toronto, Ontario; † Southlake Regional Health Centre, Newmarket, Ontario.; ‡ McMaster University/Cancer Care Ontario Program in Evidence-Based Care, Hamilton, Ontario.; § Cancer Care Ontario, Toronto, Ontario.; || Department of Radiation Oncology, Princess Margaret Hospital, Toronto, Ontario

**Keywords:** Practice guideline, depression, treatment, cancer

## Abstract

**Question:**

What is the efficacy of pharmacologic and non-pharmacologic treatments for major depression and other depressive disorders in cancer populations?

**Perspectives:**

Depression occurs at an increased rate in medically ill populations, including patients with cancer. In the general population, depression has been shown to be responsive to structured forms of psychotherapy and to pharmacologic interventions. The Supportive Care Guidelines Group conducted a systematic review of the evidence for the effectiveness of those therapies in patients with depression and cancer and developed the present clinical practice guideline based on that review and on expert consensus.

**Outcomes:**

Outcomes of interest included symptomatic response to treatment, discontinuation rate of treatment, adverse effects, and quality of life.

**Methodology:**

Clinical recommendations were developed by the Supportive Care Guidelines Group based on a systematic review of the published literature through June 2005, feedback obtained from Ontario health care providers on the draft recommendations, the Report Approval Panel (rap) of Cancer Care Ontario’s Program in Evidence-Based Care, and expert consensus.

**Results:**

The systematic review of the literature included eleven trials (seven of pharmacologic agents and four of non-pharmacologic interventions). Feedback received from 44 responding health care providers and the rap on the draft recommendations was addressed and documented in the guideline.

Among providers, 82% agreed with the draft recommendations as stated, 68% agreed that the report should be approved as a practice guideline, and 73% indicated that they would be likely to use the guideline in their own practice.

**Practice Guideline:**

These recommendations apply to adult cancer patients with a diagnosis of major depression or other non-bipolar depressive disorders. They do not address the treatment of non-syndromal depressive symptoms, for which specific antidepressant treatment is not usually indicated. The guideline is intended both for oncology health professionals and for mental health professionals engaged in the treatment of cancer patients. Expert consensus was central to the development of the guideline recommendations because of limited evidence in cancer patients.

**Recommendations:**

Treatment of pain and other reversible physical symptoms should be instituted before or with initiation of specific antidepressant treatment.

Antidepressant medications should be considered for the treatment of moderate-to-severe major depression in cancer patients. Current evidence does not support the relative superiority of one pharmacologic treatment over another, nor the superiority of pharmacologic treatment over psychosocial interventions. The choice of an antidepressant should be informed by individual medication and patient factors: the side effect profiles of the medication, tolerability of treatment (including the potential for interaction with other current medications), response to prior treatment, and patient preference.

Cancer patients diagnosed with major depression may benefit from a combined modality approach that includes both psychosocial and pharmacologic interventions. Psychosocial treatment approaches that may be of value include those that provide information and support and those that address any combination of emotional, cognitive, and behavioural factors.

**Qualifying Statements:**

Referral to a mental health specialist is appropriate when the diagnosis of depression is unclear, when the syndrome is severe, when patients do not respond to treatment, or when other complicating factors that may affect the choice of treatment are present.

Although care has been taken in the preparation of the information contained in this guideline, any person seeking to apply or to consult the guideline is expected to use independent medical judgment in the context of individual clinical circumstances or to seek out the supervision of a qualified clinician.

## 1. QUESTION

What is the efficacy of pharmacologic and non-pharmacologic treatments for major depression and other depressive disorders in cancer populations?

Outcomes of interest included symptomatic response to treatment, discontinuation rate of treatment, adverse effects, and quality of life.

## 2. CHOICE OF TOPIC AND RATIONALE

Major depression occurs in 14%–16% of cancer or palliative care patients 1,2, a rate approximately twice to four times that found in the general population 3. Individuals with serious medical conditions, including cancer, are at increased risk for persistent depressive symptoms and disorders, which are associated with significant disability 4–7. The diagnosis of major depression may be partially confounded by symptoms related to the physical effects of cancer and by “realistic” feelings of sadness, although evidence suggests that a valid diagnosis can nevertheless be made in this context 8.

Depressive disorders in the general population have been shown to be highly responsive to structured forms of psychotherapy and to pharmacologic interventions 9–11. Some studies have shown that the combination of pharmacotherapy and psychotherapy is more effective in the treatment of chronic and more severe forms of depression than is either modality alone 10,12.

Guidelines for the treatment of major depression have been published by a variety of organizations, including the National Institute for Clinical Excellence 13, the American Psychiatric Association 14, and the Canadian Psychiatric Association 15. Much of the associated evidence and the guideline recommendations may be assumed to be applicable to cancer patients; however, certain factors complicate the treatment of depressive disorders in that population, including diagnostic overlap of the symptoms of depression with those of cancer and higher rates of side effects related to medication and treatment withdrawal 8. Treatment evaluation may also be complex because of comorbid factors that contribute to depression in medical populations and that require prior or simultaneous treatment. For example, cancer pain is associated with the development and exacerbation of psychological distress, including depression 16–19 and hopelessness 20. The assessment and treatment of pain may be an essential step when pain and mood disturbance coexist 21.

Research on the effectiveness and tolerability of antidepressant treatment in cancer patients with depressive disorders is relevant because of the potential for depression in this population to be associated with various drug–drug interactions and because of the potential effect of cancer on treatment side effects, continuation rates, and outcomes. The Supportive Care Guidelines Group (scgg) therefore conducted a systematic review of the evidence on the efficacy of pharmacologic and non-pharmacologic treatments for major depression and other depressive disorders in cancer populations, and developed the present clinical practice guideline based on that review and on expert consensus.

## 3. METHODS

### 3.1 Development of the Systematic Review

The scgg of Cancer Care Ontario’s Program in Evidence-Based Care (pebc) comprises medical, radiation, and surgical oncologists; psychiatrists; palliative care physicians; nurses; radiation therapists; methodologists; administrators; a psychologist; and an anesthetist. The pebc is sponsored by, but is editorially independent of, Cancer Care Ontario and the Ontario Ministry of Health and Long-Term Care.

In 2005, a working group of the scgg conducted a systematic review of the evidence for pharmacologic and non-pharmacologic treatment of depression in cancer patients. That review included literature published through June 2005, and the sources searched included medline, embase, cinahl, Psycinfo, and the Cochrane Library. Based on predefined criteria, comparative studies of treatments for depression in cancer patients were selected for review by two scgg members. The studies were evaluated and summarized by the working group, and the completed systematic review was approved by the full scgg 22.

### 3.2 Development of the Clinical Practice Guideline

Based on the completed systematic review 22 and expert consensus, the working group used the methods of the practice guidelines development cycle 23 to draft a clinical practice guideline. The full scgg reviewed and approved the draft guideline. The draft guideline and systematic review, together with a structured survey, were distributed for external feedback to 236 health care providers in Ontario, including 101 psychiatrists, 40 medical oncologists, 41 pharmacists, 39 nurses, and 15 palliative care physicians. The survey consisted of items evaluating the methods, results, and discussion used to inform the draft guideline recommendations, and questions concerning whether the recommendations should be approved as a practice guideline. Written comments were also invited. The survey was mailed to the recipients over a period of 4 months (September through December 2005), with follow-up reminders sent at 2 weeks (postcard) and 4 weeks (complete package mailed again). The working group reviewed the provider feedback, documented the results of the feedback in the final practice guideline, and revised the guideline accordingly.

In addition, the revised guideline and systematic review were reviewed and approved by the full scgg and the pebc Report Approval Panel (rap) in October 2006. The rap consists of two members, including an oncologist, with expertise in clinical and methodologic issues. The completed guideline is intended to promote evidence-based practice and will be posted on the Cancer Care Ontario Web site (www.cancercare.on.ca), together with other guidelines produced by the pebc. The guideline will undergo periodic review and will be revised as new evidence becomes available.

## 4. RESULTS

### 4.1 Systematic Review of the Evidence

Evidence for the effectiveness of pharmacologic and non-pharmacologic interventions in the treatment of cancer patients with depressive disorders was limited. One systematic review 24, seven trials of pharmacologic agents 25–31, and four trials of non-pharmacologic interventions 32–35 were identified as relevant. Three of the eleven trials included only patients diagnosed with major depression through structured diagnostic interview. The remaining eight trials included patients with depressive symptoms above a predefined cut-off score determined using a validated assessment tool. The treatment period and follow-up was short in the trials of pharmacologic treatments (10 days to 12 weeks), which limits the conclusions that can be reached regarding long-term treatment.

The systematic review of twenty-four studies in cancer patients (six focused on antidepressant agents and eighteen on psychosocial interventions) found limited evidence in favour of both treatments 24. However, few studies in the review focused on patients diagnosed with a depressive disorder; most were studies of prevention or included patients with mild depressive symptoms.

Two pharmacologic trials comparing mianserin to placebo detected a significant benefit with treatment 25,31. In another trial, alprazolam was found to be superior to progressive muscle relaxation in reducing depressive symptoms 27. Four of the pharmacologic trials found no significant difference between groups on a measure of depression 26,28–30. Two of those trials compared low-dose fluoxetine to placebo 26,30, one compared fluoxetine to desipramine 28, and one compared paroxetine to amitriptyline 29. In the latter two studies, significant pre–post treatment effects occurred for both active comparators, but the significance of these findings in the absence of placebo comparators is limited. Only one of the pharmacologic trials assessed outcome based on remission of depressive symptoms to within the normal range as opposed to response, which is a less stringent outcome.

Two of the four trials that assessed non-pharmacologic therapies for the management of depression found a significant difference between treatment groups. One trial found a benefit in using a multi-component nurse-delivered intervention 35, with a reduction in the number of patients diagnosed with major depression. The other positive trial found the use of an orientation program to be beneficial in reducing depressive symptoms 34. In both trials, the control group received usual care. Neither group psychotherapy nor adjuvant psychological therapy (cognitive behavioural therapy) was found to significantly reduce depressive symptoms in the other two non-pharmacologic trials 32,33.

### 4.2 Practitioner Feedback

The draft clinical practice guideline and systematic review were circulated to 236 health care providers in Ontario for review and feedback. From among the 236 surveys mailed, 75 responses were received (32% response rate). Of the respondents, 44 individuals—including 13 medical oncologists, 11 nurses, 10 psychiatrists, 7 palliative care physicians, and 3 pharmacists—indicated that the report was relevant to their clinical practice, and they completed the survey. Table I shows key results of the external review survey, summarized by respondent discipline.

Among all respondents, 36 (82%) agreed with the draft recommendations as stated (7% neither agreed nor disagreed); 30 (68%) agreed that the report should be approved as a practice guideline (14% neither agreed nor disagreed); and 32 (73%) indicated that they would be likely to use the guideline in their own practice (18% were unsure). Written comments related to the content of the report were provided by 21 respondents (48%). Several respondents thought that the systematic review was well done and that the guideline was an important initiative. The main points contained in the remaining written comments are summarized in the subsection that follows.

#### 4.2.1 Comments on the Recommendations

Based on the expert consensus of the group, the draft guideline included recommendations for specific pharmacologic agents. Some respondents commented on the lack of evidence for most of the agents, noting the limitation of having only two psychiatrists within the group as a source of consensus. The sub-recommendation on “treatment of pain” was considered too rigid by some respondents; they indicated that concurrent use of pain management and antidepressant medications is sometimes appropriate. One respondent suggested that tricyclic antidepressants could be mentioned within the recommendations, if only to discourage their use in major depression unless as an adjuvant agent in analgesia. It was also suggested that the guideline could include discussions concerning when to defer to a specialist (psychologist, social worker, psychiatrist) and the issues involved in the collaborative care of cancer patients.

#### 4.2.2 Comments on the Evidence

Survey respondents commented on a number of studies that they felt were of interest or that should be included in the report, and they mentioned a need for clearer identification of the evidence for treatment of depression in other medically ill populations.

#### 4.2.3 Other Comments

A number of respondents commented on the use of screening for depression. One felt that the review should include stronger emphasis on the need for systematic screening, although such screening may require reorganization of resources. One suggested that a recommended screening tool for depression in cancer patients would be useful, and one indicated that the use of screening tools is of limited benefit in patients with depersonalization, who benefit little from the use of antidepressants. Two respondents commented on the need for family doctors, oncologists, and other specialists to become involved in the assessment and treatment of depression in cancer patients so that treatment is actually offered where indicated. One respondent remarked that support from psychiatrists would be needed for such an effort, and one suggested that support for the families and caregivers of patients should also be considered, because they are at higher risk for depression.

### 4.3 Guideline Group and PEBC Report Approval Panel

The final guideline was reviewed and approved by the rap and the full scgg in October 2006. Key issues raised by the rap included a need for clarification on the intended provider audience for the report and consideration of the presentation of the information for the specific audience. Also, given the limited evidence for treatment options in cancer patients, they requested further discussion of the evidence for treatment effectiveness in non-cancer populations. The latter point was also raised by participants during the external review process. Similarly, the full scgg commented on the need for greater emphasis in the guideline on the mixed evidence regarding the impact of screening for depression on patient outcomes, an issue that was also raised during external review.

## 5. DISCUSSION

During initial discussions on the development of a guideline for the treatment of depressive disorders in cancer patients, the scgg members raised three questions:

- First, how valid and reliable is the diagnosis of depressive disorder in cancer patients, and what is the prevalence and course of this condition in the population with cancer?
- To what extent do systematic reviews, meta-analyses, and randomized controlled trials confirm the efficacy of antidepressant treatments in the population with cancer?
- Do guidelines for the treatment of depressive disorders in cancer patients and in other populations already exist?

These questions directed the discussion toward development of a guideline, with the group deciding to focus on the treatment of depression. Screening and diagnosis of depression in cancer patients would be a topic for future consideration.

The scgg found that the evidence suggests that a valid and reliable diagnosis of major depression can be made in the cancer population despite the overlap of symptoms of depression with those of cancer and its treatment. Depressive symptoms have been shown to persist and to be associated with significant morbidity in medically ill populations. Because milder depressive symptoms are a common non-specific manifestation of distress in cancer patients, a group decision was made to focus on the syndrome of major depression, for which specific interventions have been developed.

The “gold standard” for the diagnosis of major depression is a structured diagnostic interview; however, because of limited evidence in the population under consideration, the guideline was subsequently expanded to include studies of patients with depressive symptoms above a predefined cut-off point on a validated depression assessment scale. In addition, given the available evidence, the group decided to present the evidence in two distinct categories: pharmacologic trials, and trials of non-pharmacologic interventions.

Following review of the draft guideline by Ontario health care providers, the full scgg, and the rap, a number of revisions were incorporated into the guideline. The working group acknowledged the limitations of the current evidence, considered the disparity between available research evidence and current practice, and discussed the value of providing guidance for clinicians on the use of specific pharmacologic agents in the absence of evidence. Although opinions varied, the group agreed that recommendations for specific antidepressants in the absence of evidence would not be provided within the current guideline. Instead, recommendations on future research to evaluate specific antidepressants were provided.

The working group discussed referral of patients to a specialist (psychologist, social worker, psychiatrist) and the issues involved in the collaborative care of patients. They emphasized the need for all health care providers to be alert to signs and symptoms of depression in cancer patients, and they agreed that referral to a mental health specialist is most appropriate when the diagnosis of depression is unclear, when the syndrome is severe, when patients do not respond to treatment, or when other complicating factors that may affect the choice of treatment are present. A qualifying statement to this effect was added to the guideline.

With regard to the recommendation on treatment of pain, the group felt that the current recommendation did not preclude concomitant pain management and depression treatment and did not require revision. Similarly, at the present time, they chose not to add a recommendation explicitly discouraging the use of tricyclic antidepressants, because although newer classes of antidepressants have fewer side effects than tricyclics, the new agents have not been shown to be more effective than tricyclics.

The working group considered the additional references identified by the external reviewers and, although of interest, none of those studies met the predefined inclusion criteria for the systematic review of the evidence. However, the need for clearer identification of evidence for treatment of depression in other medically ill populations was raised by a number of external reviewers and by the rap. As a result, discussion of this evidence and its relevance to the treatment of depression in cancer patients was expanded within the systematic review, although this change did not require a change in the guideline recommendations.

Another key issue that was raised by external reviewers and by some scgg members was the evidence related to screening for depression. Evidence from the general population and from patients with other medical conditions suggests that screening for depression may result in increased recognition of depression; however, evidence for a positive impact of such screening on the management of depression or on outcome has been mixed 36,37. Because a systematic review of the evidence on screening was not conducted for this guideline, no recommendations could be made on the need for systematic screening programs at this time. Instead, when depression is detected, the guideline emphasizes that appropriate treatment, follow-up, and referral should be undertaken as necessary. The evaluation of depression screening tools (suggested by one external reviewer) is beyond the scope of the current guideline and will be considered for future report development.

The current guideline is intended both for oncology health professionals and for mental health professionals engaged in the treatment of cancer patients, and in response to the rap feedback, a section on the target provider population was added to the guideline. Although mental health professionals may have more expertise in the use of screening tools for depression, it is not clear that the method of detection or presentation of depression affects treatment outcomes, and therefore this guideline is also considered appropriate for oncology health professionals. The need for a range of health care professionals to become involved in the assessment and treatment of depression in cancer patients is acknowledged. The hope is that this report will provide guidance regarding treatment options. The importance of the broader issue of assessment and treatment of families and caregivers of cancer patients is recognized; however, broader guidelines for treatment of depression in non-medical populations would also apply in that population.

## 6. PRACTICE GUIDELINE

Clear evidence derived from randomized controlled trials in cancer patients that could be used to inform conclusions is absent. The recommendations that follow therefore reflect the expert consensus of the scgg members, informed by the evidence reviewed and by the feedback received from Ontario health care providers and the pebc rap.

### 6.1 Target Patient Population

These recommendations apply to adult cancer patients with a diagnosis of major depression or other non-bipolar depressive disorders. They do not address the treatment of non-syndromal depressive symptoms, for which specific antidepressant treatment is not usually indicated. Such symptoms are frequent as non-specific manifestations of distress or in association with pain or other suffering. For the purposes of this guideline, conclusions are based on evidence from studies in two categories of patients:

- Patients diagnosed with major depression by a structured diagnostic interview. This method is the “gold standard” for a diagnosis of a depressive disorder.
- Patients with depressive symptoms scoring more than 14 on the first 17 items of the Hamilton Depression Rating Scale, 8 or higher on the Hospital Anxiety and Depression Scale, or above the equivalent cut-off on another validated assessment scale, realizing the these measures were developed to assess symptoms. They are used for screening, but they are less stringent methods for diagnosing depressive disorders because they may be associated with false positives and false negatives. Some (but not all) of these patients may have been suffering from major depression, dysthymic disorder, adjustment disorder with depressed mood, or minor depression.

### 6.2 Target Provider Population

The guideline is intended both for oncology health professionals and for mental health professionals engaged in the treatment of cancer patients. Referral to a mental health specialist may be valuable for cancer patients diagnosed with major depression, but such a referral may not always be feasible. The rate of detection of depressive disorders in this and other populations is increased by the use of screening measures, but there is no evidence that the nature of the disorders or their response to treatment varies by the method of detection or presentation.

### 6.3 Recommendations

Treatment of pain and other reversible physical symptoms should be instituted before or with initiation of specific antidepressant treatment.

Antidepressant medications should be considered for the treatment of moderate-to-severe major depression in cancer patients. Current evidence does not support the relative superiority of one pharmacologic treatment over another, nor the superiority of pharmacologic treatment over psychosocial interventions. The choice of an antidepressant should be informed by individual medication and patient factors: the side effect profiles of the medication, tolerability of treatment (including the potential for interaction with other current medications), response to prior treatment, and patient preference.

Cancer patients diagnosed with major depression may benefit from a combined modality approach that includes both psychosocial and pharmacologic interventions. Psychosocial treatment approaches that may be of value include those that provide information and support and those that address any combination of emotional, cognitive, and behavioural factors.

### 6.4 Qualifying Statements

Referral to a mental health specialist is appropriate when the diagnosis of depression is unclear, when the syndrome is severe, when patients do not respond to treatment, or when other complicating factors that may affect the choice of treatment are present.

Although care has been taken in the preparation of the information contained in this guideline, any person seeking to apply or to consult the guideline is expected to use independent medical judgment in the context of individual clinical circumstances or to seek out the supervision of a qualified clinician.

## 7. FUTURE RESEARCH

Large multicentre studies of patients with histologically similar cancers are required to evaluate the efficacy of antidepressant interventions, including the relative benefit of psychological versus pharmacologic interventions in specific cancers, in which medication side effect profiles, physical symptoms and psychosocial problems, and the efficacy of specific antidepressant medications may vary. Indications for specific medications that deserve further investigation include the potential value of mirtazapine in the treatment of mood disorders accompanied by nausea, weight loss, insomnia, or anxiety; the use of dual-action antidepressants such as mirtazapine, venlafaxine, and duloxetine in the treatment of co-morbid pain and depression; and the use of sustained release bupropion for cancer patients with significant symptoms of depression and fatigue.

Further studies are needed to evaluate the relative effectiveness and tolerability of newer antidepressant treatments and the use of combination strategies for treatment-resistant depressive disorders. The latter potentially include the use of two antidepressant medications used in combination and the use of antidepressants combined with either lithium or atypical antipsychotics.

Research is needed to identify strategies to improve the rates of detection and treatment completion in cancer patients with depressive disorders.

## 8. PRACTICE GUIDELINE DATE

The present clinical practice guideline report is based on work completed in October 2006. All approved pebc reports are updated periodically and posted on the Cancer Care Ontario Web site (www.cancercare.on.ca/index_practiceGuidelines.htm).

## Figures and Tables

**TABLE I:** Responses to eight items on the external review survey

Item	Responders	Strongly agree or agree	[*n* (%)] a Neither agree nor disagree	Strongly disagree or disagree
The rationale for developing a guideline, as stated in the Introduction section of the Systematic Review, is clear.	Psychiatrists	9 (90)	1 (10)	0
	Medical oncologists ^b1^	11 (85)	1 (8)	0
	Nurses	9 (82)	0	2 (18)
	Palliative care physicians	7 (100)	0	0
	Pharmacists	3 (100)	0	0
There is a need for a guideline on this topic.	Psychiatrists	10 (100)	0	0
	Medical oncologists ^b1^	9 (69)	3 (23)	0
	Nurses	9 (82)	0	2 (18)
	Palliative care physicians	7 (100)	0	0
	Pharmacists	3 (100)	0	0
The literature search is relevant and complete.	Psychiatrists ^b1^	7 (70)	2 (20)	0
	Medical oncologists ^b1^	10 (77)	2 (15)	0
	Nurses ^b1^	6 (55)	2 (18)	2 (18)
	Palliative care physicians	3 (43)	4 (57)	0
	Pharmacists	1 (33)	1 (33)	1 (33)
The results of the trials described in the Systematic Review are interpreted according to my understanding of the data.	Psychiatrists	8 (80)	1 (10)	1 (10)
	Medical oncologists ^b1^	7 (54)	5 (38)	0
	Nurses	8 (73)	1 (9)	2 (18)
	Palliative care physicians ^b2^	4 (57)	1 (14)	0
	Pharmacists	3 (100)	0	0
The draft recommendations in the Clinical Practice Guideline are clear.	Psychiatrists	10 (100)	0	0
	Medical oncologists ^b1^	12 (92)	0	0
	Nurses	9 (82)	0	2 (18)
	Palliative care physicians ^b1^	4 (57)	0	2 (29)
	Pharmacists	3 (100)	0	0
I agree with the draft recommendations as stated.	Psychiatrists	9 (90)	0	1 (10)
	Medical oncologists ^b1^	11 (85)	1 (8)	0
	Nurses	8 (73)	2 (18)	1 (9)
	Palliative care physicians ^b1^	5 (71)	0	1 (14)
	Pharmacists	3 (100)	0	0
This report should be approved as a practice guideline.	Psychiatrists	7 (70)	2 (20)	1 (10)
	Medical oncologists ^b1^	11 (85)	1 (8)	0
	Nurses ^b1^	6 (55)	2 (18)	2 (18)
	Palliative care physicians	4 (57)	1 (14)	2 (28)
	Pharmacists	2 (67)	0	1 (33)

		Very likely or likely	Unsure	Not at all likely or unlikely

If this report were to become a practice guideline, how likely would you be to make use of it in your own practice?	Psychiatrists	6 (60)	3 (30)	1 (10)
	Medical oncologists ^b1^	10 (77)	2 (15)	0
	Nurses ^b1^	8 (73)	2 (18)	0
	Palliative care physicians	5 (71)	1 (14)	1 (14)
	Pharmacists	3 (100)	0	0

a Percentages may not add to 100% because of rounding.

bx Some responders (*n* = x) did not answer this question.
